# SARS-CoV-2 accessory protein ORF8 is secreted extracellularly as a glycoprotein homodimer

**DOI:** 10.1016/j.jbc.2022.101724

**Published:** 2022-02-11

**Authors:** Kazuhiro Matsuoka, Nobuhiko Imahashi, Miki Ohno, Hirotaka Ode, Yoshihiro Nakata, Mai Kubota, Atsuko Sugimoto, Mayumi Imahashi, Yoshiyuki Yokomaku, Yasumasa Iwatani

**Affiliations:** 1 Department of Infectious Diseases and Immunology, Clinical Research Center, National Hospital Organization Nagoya Medical Center, Nagoya, Aichi, Japan; 2 Department of Hematology, National Hospital Organization Nagoya Medical Center, Nagoya, Aichi, Japan; 3 Department of AIDS Research, Nagoya University Graduate School of Medicine, Nagoya, Aichi, Japan

**Keywords:** SARS-CoV-2, COVID-19, ORF8, glycosylation, secretory protein, 2-ME, 2-mercaptoethanol, ACE2, angiotensin-converting enzyme 2, CoV-2 S, SARS-CoV-2 spike, ER, endoplasmic reticulum, FL, full-length, IL17RA, interleukin(IL)-17 receptor A, MDM, monocyte-derived macrophage, MHC-I, major histocompatibility complex class Ι, ORF8, open reading frame 8, PBS, phosphate-buffered saline, SARS-CoV-2, severe acute respiratory syndrome coronavirus 2, sup, supernatant, WT, wild-type

## Abstract

ORF8 is an accessory protein encoded by severe acute respiratory syndrome coronavirus 2 (SARS-CoV-2). Consensus regarding the biological functions of ORF8 is lacking, largely because the fundamental characteristics of this protein in cells have not been determined. To clarify these features, we herein established an ORF8 expression system in 293T cells. Using this system, approximately 41% of the ORF8 expressed in 293T cells were secreted extracellularly as a glycoprotein homodimer with inter/intramolecular disulfide bonds. Intracellular ORF8 was sensitive to the glycosidase Endo H, whereas the secreted portion was Endo-H-resistant, suggesting that secretion occurs *via* a conventional pathway. Additionally, immunoblotting analysis showed that the total amounts of the major histocompatibility complex class Ι (MHC-I), angiotensin-converting enzyme 2 (ACE2), and SARS-CoV-2 spike (CoV-2 S) proteins coexpressed in cells were not changed by the increased ORF8 expression, although FACS analysis revealed that the expression of the cell surface MHC-I protein, but not that of ACE2 and CoV-2 S proteins, was reduced by ORF8 expression. Finally, we demonstrate by RNA-seq analysis that ORF8 had no significant stimulatory effects in human primary monocyte-derived macrophages (MDMs). Taken together, our results provide fundamental evidence that the ORF8 glycoprotein acts as a secreted homodimer, and its functions are likely associated with the intracellular transport and/or extracellular signaling in SARS-CoV-2 infection.

Despite extensive studies on severe acute respiratory syndrome coronavirus 2 (SARS-CoV-2), the functions of viral gene products have not been characterized, hampering our understanding of the biology of coronavirus disease 2019 (COVID-19). To further understand the functions of its viral proteins, it is necessary to investigate its biochemical properties and functions in cells using appropriate systems. Except for that of the SARS-CoV-2 spike protein (CoV-2 S), the functions of viral accessory proteins have not yet been widely studied.

Although the viral genes of severe acute respiratory syndrome coronavirus (referred to as SARS-CoV-1 in this study) and SARS-CoV-2 are relatively conserved, many genetic mutations and deletions/insertions are observed in the locus near the *open reading frame 8* (*orf8*) genes among betacoronaviruses (1, 2, 3, 4, 5, 6, 7, 8, 9, 10). Therefore, the ORF8 protein is regarded as one of the most rapidly evolving betacoronavirus proteins (4, 6, 7, 8). In addition, the ORF8 amino acid sequences tend to be mutated in epidemics and during adaptation to novel hosts, suggesting that they play an important role in pathogenicity (2, 4, 5, 11). In contrast, the expression of the ORF8 protein does not appear to be strictly essential for the *in vitro* replication of SARS-CoV-1 and SARS-CoV-2 (12, 13). In the case of SARS-CoV-1, a 29-nucleotide deletion that occurred early in the human-to-human transmission of the virus in 2002 split *orf8* into *orf8a* and *orf8b*, which correlated with attenuation of the disease (3, 6, 14, 15, 16). Furthermore, during the SARS-CoV-1 epidemic, a strain of SARS-CoV-1 with an 82-nucleotide deletion and a large-scale deletion in the *orf8* gene was also detected (3). Moreover, a deletion of 382 bases (Δ382) in SARS-CoV-2, detected in Singapore, was found to correlate with milder disease and lower incidence of hypoxia (11). However, the variant of concern (VOC), the alpha strain (B.1.1.7) that carries a nonsense mutation in *orf8* gene (Q27∗) was first detected in the United Kingdom in late 2020 and caused a worldwide pandemic that included many severe cases (17, 18, 19). The Q27∗ of the alpha strain was first thought to be a mutation that splits or inactivates the ORF8 protein (11, 12, 20, 21).

SARS-CoV-2 ORF8 is a 121 amino acid protein that presumably has an N-terminal signal sequence and a glycosylation motif, asparagine-X-serine/threonine (X is any amino acid except proline), as determined by *in silico* analysis (8, 22). Previous studies showed that in cells, the ORF8 proteins of both SARS-CoV-1 and SARS-CoV-2 localize to the endoplasmic reticulum (ER) and that the SARS-CoV-2 ORF8 protein interacts with a variety of host proteins in the ER lumen, including the factors involved in ER-associated degradation and immunity (23, 24, 25). Because anti-ORF8 antibodies are produced in patients infected with SARS-CoV-2, the ORF8 protein may be secreted beyond the ER (22). Several other functions of the ORF8 protein have also been reported thus far. The SARS-CoV-2 ORF8 protein has been reported to induce ER stress (26, 27), antagonize the interferon (IFN) signaling pathway (26, 28, 29), downregulate major histocompatibility complex class Ι (MHC-I) molecules in cells (30), and interact with transforming growth factor-beta 2 (TGF-β2) to participate in the TGF-β1 signaling cascade (25). In addition, the ORF8 protein was found to promote the expression of inflammatory factors by interacting with host interleukin(IL)-17 receptor A (IL17RA) (31). However, consensus on these phenomena is limited yet, and the fundamental functions of the SARS-CoV-2 ORF8 protein must be elucidated (9).

One of the most critical points for elucidating its functions is determining its biochemical characteristics in cell using appropriate systems. For example, the molecular size of the ORF8 protein expressed in cells differs among previous reports (22, 23, 25, 27, 28, 30, 31). This prompted us to assess ORF8 protein expression and determine its characteristics in cells. First, when expressed with the ORF8 expression plasmid encoding viral *orf8* cDNA, the mRNA expressed in cells was unexpectedly spliced at cryptic splice sites, which resulted in the production of an ORF8 protein smaller than the theoretical size. Optimization of codons and removal of cryptic splice sites enabled us to build a new ORF8 expression system that allowed the production of mRNA and protein at the appropriate sizes. Using this system, we found that approximately 41% of SARS-CoV-2 ORF8 was secreted out of the cell as a homodimeric glycoprotein with intramolecular disulfide bonds. In addition, we observed ORF8-dependent downregulation of MHC-I. In contrast, stimulatory effects of the secreted ORF8 protein were not detected in primary human monocyte-derived macrophages (MDMs). These studies provide a basic system to further identify the fundamental functions of the ORF8 protein in SARS-CoV-2 infection.

## Results

### Cryptic splice sites in the ORF8 coding region are used upon orf8 cDNA expression

**Figure 1:**
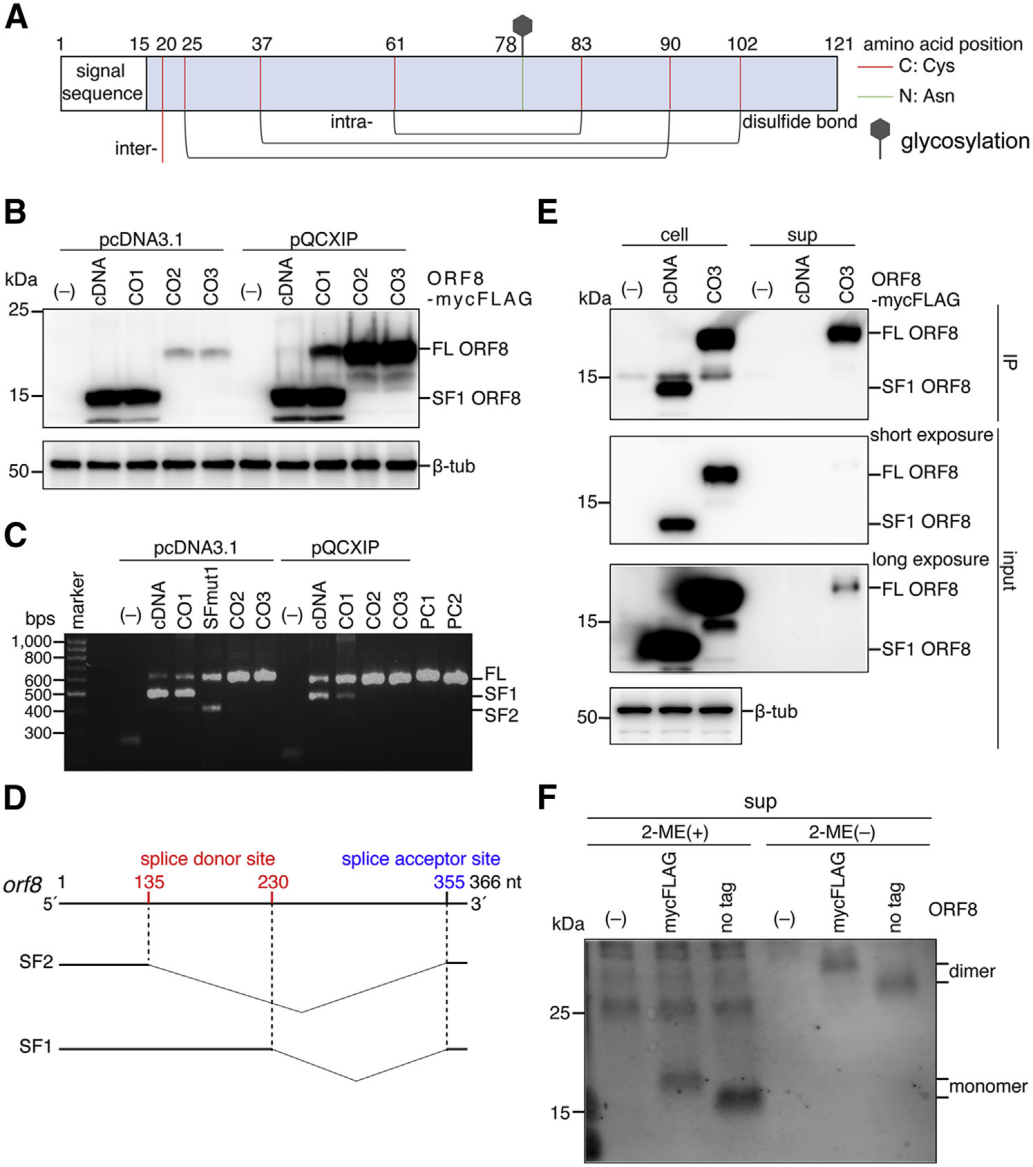
**Expression of the SARS-CoV-2 ORF8 protein in 293T cells.***A*, schematic representation of the SARS-CoV-2 ORF8 protein. ORF8 is presumably a secretory protein with an N-terminal signal peptide and a glycosylation site at N78 (*green line*). Cysteine residues involved in the formation of intermolecular (inter) and intramolecular (intra) disulfide bonds are indicated by the *red line*. *B*, Western blot analysis. Briefly, 293T cells were transfected with C-terminally myc-FLAG-tagged ORF8 expression plasmids containing no ORF8 gene (–), ORF8 cDNA (cDNA), codon optimized ORF8 gene 1 (CO1), or the additionally optimized ORF8 gene 2 (CO2) or 3 (CO3). The pcDNA 3.1 or pQCXIP expression vector was used for transient expression. The proteins were detected by Western blotting using anti-FLAG tag mAb and anti-β-tub antibody. The bands corresponding to “FL” ORF8-mycFLAG (FL ORF8) and “spliced form 1” ORF8-mycFLAG (SF1 ORF8) are indicated. *C*, analysis of mRNA in the transfected 293T cells. DNA fragments amplified by RT-PCR were separated by electrophoresis and stained with ethidium bromide. The fragments amplified from pcDNA 3.1 ORF8-mycFLAG (PC1: 678 bps) and pQCXIP 3.1 ORF8-mycFLAG (PC2: 604 bps) were used as positive controls. *D*, schematic representation of the cryptic splice sites in the ORF8 cDNA. Based on the fragment sequences, two major sliced forms were found: spliced form 1 (SF1) and spliced form 2 (SF2). The “SFmut1” is the ORF8 cDNA of which the second cryptic donor site (230 nt) was mutated. The major cryptic splice sites in the open reading frame are indicated. *E*, analysis by pull-down assay. ORF8-mycFLAG proteins were expressed in 293T cells using ORF8-mycFLAG expression plasmids, cDNA-derived ORF8 (cDNA) and optimized ORF8-mycFLAG (CO3). ORF8 proteins in cells (cell) and supernatants (sup) were immunoprecipitated with anti-FLAG mAb beads. The immunoprecipitated (IP) and input fractions were analyzed by Western blot using anti-FLAG mAb and β-tub antibody. *F*, analysis of ORF8 with or without 2-ME. The gene-optimized ORF8 (CO3) with or without mycFLAG was transiently expressed in 293T cells. The samples treated with and without 2-ME were separated by SDS-PAGE and analyzed by Western blot with an anti-SARS-CoV-2 ORF8 rabbit polyclonal antibody. 2-ME, 2-mercaptoethanol; ORF8, open reading frame 8; SARS-CoV-2, severe acute respiratory syndrome coronavirus 2.

SARS-CoV-2 ORF8 is a putative secretory protein comprised of 121 amino acids that contains an N-terminal signal sequence and a glycosylation site at the 78th (N78) position (Fig. 1*A*). Based on the crystal structure obtained by oxidative refolding of the recombinant ORF8 protein from *E. coli*, ORF8 forms a homodimer with three pairs of intramolecular disulfide bonds per protomer and one pair of intermolecular disulfide bonds *via* cysteine 20 (C20) (Fig. 1*A*) (32). To characterize the biological features of ORF8 in cells, we first cloned SARS-CoV-2 ORF8 cDNA directly from viral RNA (vRNA) and constructed an expression plasmid for the ORF8 protein with a mycFLAG tag at the C-terminus (ORF8-mycFLAG). ORF8-mycFLAG-derived cDNA was overexpressed in Human embryonic kidney 293T (293T) cells and analyzed by Western blot. As shown in Figure 1*B*, the band of ORF8-mycFLAG migrated at 14.1 kDa, slightly faster than that of the theoretically sized protein (>15.8 kDa as a glycoprotein of the peptide 16–121), suggesting abnormal ORF8 expression through this system (Fig. 1*B*). Because SARS-CoV-2 is a positive-sense single-stranded RNA virus, both vRNA replication and viral protein synthesis take place in the cytoplasmic environment. In contrast, in our overexpression system mediated by a mammalian expression vector encoding cDNA, ORF8 mRNA transcribed in the nucleus is transferred to the cytoplasm, where protein synthesis occurs. Therefore, we hypothesized that the abnormal ORF8 expression was due to unexpected splicing as cryptic splice site(s) in ORF8 RNA. To assess this possibility, we isolated total RNA from transfected 293T cells, amplified *orf8* gene products by RT-PCR, and analyzed the sequences by the Sanger method. One major spliced product SF1, but not a full-length (FL) product, was detected. SF1 had a 126-nucleotide (nt) in-frame deletion between the GU-AG splice motif. Inactivation of the splice donor site at the 230th position resulted in production of both a FL product and an alternative spliced fragment SF2 that had a 221-nt out-of-frame deletion (SF1, Fig. 1*C* and Table S1). These results indicate that upon ORF8 cDNA expression, the following cryptic splice sites in the coding region are predominantly used: two active splice donor sites (135 nt and 230 nt) and one splice acceptor site (355 nt) (Fig. 1*D*). When the other plasmid, pQCXIP, was used, FL RNA products were increased (Fig. 1*C*). Therefore, we rebuilt three ORF8 expression plasmids by removing the cryptic splice sites (CO2 and CO3), optimizing the *orf8* codons (CO1, CO2, and CO3), and inserting the genes into the retroviral vector pQCXIP. The sequences of the *orf8* genes designed herein are listed in Table S1. Then, 293T cells were transfected with each plasmid, and the ORF8 proteins were analyzed. The major bands of CO2 and CO3 were found at approximately 19.9 kDa (Fig. 1*B*). In addition, only FL RT-PCR products were detected in CO2 and CO3 (Fig. 1*C*). Therefore, pQCXIP ORF8-mycFLAG (CO3) was used in subsequent experiments for ORF8 protein expression.

### The ORF8 protein is secreted extracellularly

Based on our *in silico* analyses, the ORF8 sequences had a signal sequence cleavage site at the 15th peptide bond and no predicted ER-retention motifs (8, 22, 33), suggesting that ORF8 is a secretory protein. To confirm this possibility, we overexpressed ORF8-mycFLAG CO3 in 293T cells, and the proteins in cells and supernatants (sups) were analyzed by an immunoprecipitation assay using anti-Flag magnetic beads. As expected, ORF8-mycFLAG CO3 was detected in both cells and the culture sup, whereas SF1 ORF8 was found only in cells (Fig. 1*E*). Based on the quantification of the intensity of each band in the immunoblots, approximately 41% of the total ORF8 protein was retained in the sup fraction (Fig. S1). These results suggest that ORF8 is efficiently secreted extracellularly, although ORF8 SF1 accumulated inside of the cells and was not secreted extracellularly. We further examined the secreted ORF8 protein in the sup under reducing conditions with 2-mercaptoethanol (2-ME). In the presence of 2-ME, a specific band of the ORF8-mycFLAG protein was detected at approximately 19 kDa, which roughly corresponds to its monomer size (Fig. 1*F*). In contrast, in the absence of 2-ME, it displayed at 33 kDa, which was approximately twice the size of the monomer. These data indicate that ORF8 is secreted extracellularly as a dimer that is covalently linked *via* a disulfide bond(s). In addition, as expected, because the molecular size of the detected monomer ORF8-mycFLAG was 3.6 kDa larger than the theoretical size of the protein, the results suggest that the secreted ORF8 is likely glycosylated. Of note, these results were obtained even when using the nontagged ORF8 version (Fig. 1*F*).

### ORF8 is modified with an endoglycosidase H (Endo H)-resistant glycan chain at the N78 residue in cells

**Figure 2:**
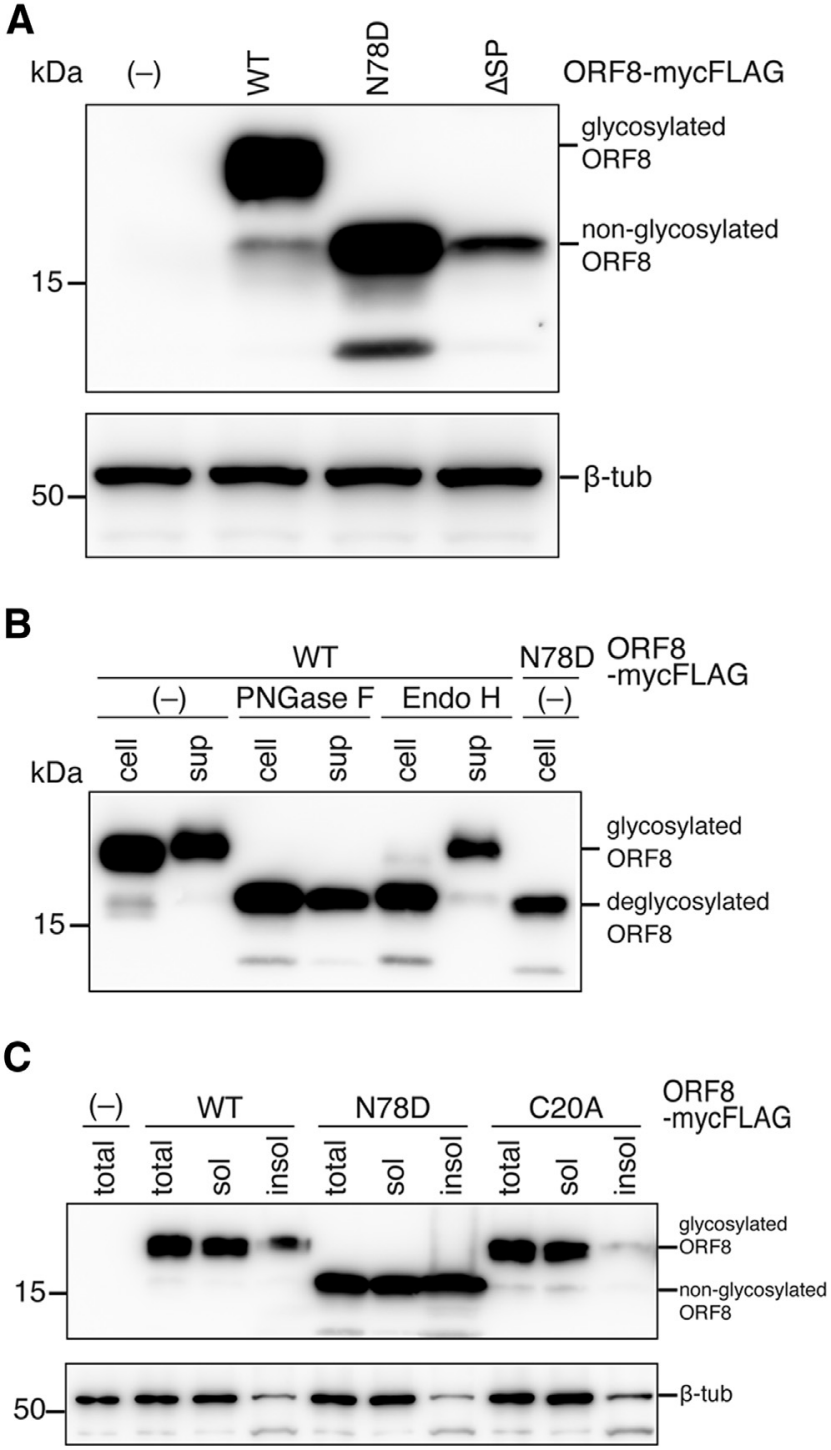
**Characterization of the N-linked glycosylation of the SARS-CoV-2 ORF8 protein.***A*, briefly, 293T cells were transfected with the wild-type (WT) or mutant ORF8-mycFLAG expression plasmid. Cell lysates were denatured under reducing conditions with 2-ME and then visualized by immunoblotting with an anti-FLAG mAb and anti-β-tub antibody. The ORF8 mutant deficient for the signal sequence (ΔSP) was used as a negative control. *B*, the Endo H and PNGase F sensitivities of the ORF8 proteins were assessed. The WT and N78D mutant were pulled down with anti-FLAG mAb beads from the cell lysate (cell) and culture supernatant (sup). The IP factions were untreated (–), or incubated with either PNGase F or Endo H glycosidase. The digested samples were subjected to immunoblot analysis using an anti-FLAG mAb. *C*, the total fraction (total), soluble fraction (sol), and insoluble fraction (insol) were analyzed by Western blot using anti-FLAG mAb and anti-β-tub antibody. 2-ME, 2-mercaptoethanol; ORF8, open reading frame 8; SARS-CoV-2, severe acute respiratory syndrome coronavirus 2.

Next, we analyzed the intracellular expression of wild-type (WT) ORF8 and two ORF8 mutants: N78D with an N-linked glycosylation site deficiency and the ORF8 ΔSP mutant with a 15-amino acid signal sequence deletion. The ΔSP mutant, which was expected to be 14.4 kDa and to localize in cytoplasm, was used as control. As shown in Figure 2*A*, the N78D band was detected at approximately 16 kDa, which was equivalent to that of ΔSP (Fig. 2*A*). These results demonstrated that the ORF8 protein is translocated into ER, followed by cleavage of the signal sequence and glycosylation at residue N78. To clarify the glycosylation type (high mannose, hybrid, or complex-type), we expressed ORF8-mycFLAG proteins in 293T cells, immunoprecipitated them using anti-Flag magnetic beads, and assessed their sensitivity to two types of glycosidases: peptide-N-glycosidase F (PNGase F) and Endo H. As shown in Figure 2*B*, the ORF8 WT protein collected from cells was sensitive to PNGase F and Endo H. In contrast, the ORF8 WT immunoprecipitated from sup was sensitive to PNGase F but resistant to Endo H (Fig. 2*B*). These results suggest that the majority of intracellular ORF8 proteins have an N-linked high mannose chain, whereas the secreted portion has a complex-type (or hybrid-type) sugar chain. Furthermore, this result suggests that ORF8 is secreted *via* the Golgi-mediated glycoprotein transport and secretion pathway.

To further understand the role of glycosylation in ORF8, we compared the levels of N78D with the WT and C20A mutant in soluble and insoluble cell fractions. The C20A mutant that is deficient for intermolecular disulfide bonds was herein used as control because glycosylation of ORF8 appeared to be independent of stabilization *via* intermolecular disulfide bonding based on information of the ORF8 crystal structure. The results showed that the levels of WT and the C20A mutant were significantly low in the insoluble fraction, although the N78D mutant was highly detected in the insoluble fraction (Fig. 2*C*). These data demonstrated that glycosylation of ORF8 at the N78 residue increases its solubility in cells.

### Glycosylation of ORF8 is required for its efficient secretion, although intermolecular disulfide bonding of the homodimer is dispensable for its secretion

**Figure 3:**
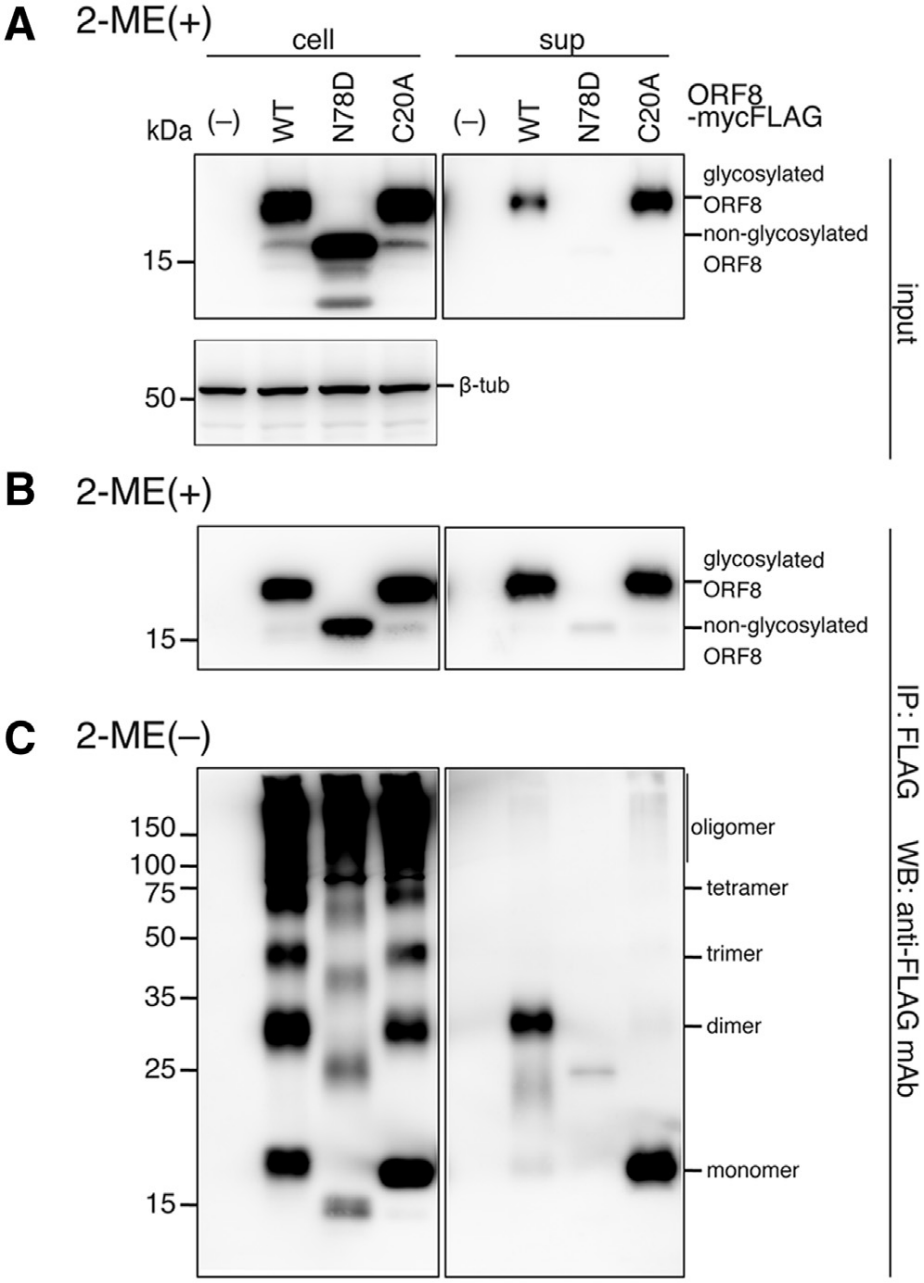
**Effects of SARS-CoV-2 ORF8 mutations at glycosylation and cysteine residues on secretion.***A*, wild-type (WT) and mutant ORF8-mycFLAG were expressed in 293T cells and separated by SDS-PAGE. Total fractions (input) treated with 2-ME were analyzed by immunoblotting using anti-FLAG mAb and anti-β-tub antibody. *B* and *C*, the immunoprecipitated (IP) fractions were prepared using anti-FLAG mAb beads and treated with 2-ME (*B*) or without 2-ME (*C*). The IP fractions were analyzed by Western blot using an anti-FLAG mAb. The approximate sizes (kDa) of the molecular weight standards for SDS-PAGE are indicated on the *left*. 2-ME, 2-mercaptoethanol; ORF8, open reading frame 8; SARS-CoV-2, severe acute respiratory syndrome coronavirus 2.

Next, to further characterize intracellular modifications of the ORF8 protein, we investigated the cellular secretions of the ORF8 N78D mutant and the C20A mutant. We expressed either of the ORF8 mutants in 293T cells, pulled down ORF8-mycFLAG proteins in cells (cell) and sups with anti-FLAG-tagged beads, and analyzed them by immunoblotting. As expected, the N78D bands in the lysate and sup migrated 3.6 kDa faster than the WT or C20A mutant in the presence of 2-ME (Fig. 3*A*), indicating that the secreted ORF8 was glycosylated at the N78 position and that the C20A mutation did not disrupt ORF8 glycosylation. In the sups, the level of ORF8 N78D was dramatically reduced compared with that of the WT and C20A (Fig. 3*A*). These results indicate that ORF8 glycosylation is critical for its efficient secretion into the cell sup. These trends were also observed in the immunoprecipitated (IP) fractions (Fig. 3*B*).

When the proteins in the lysate were separated in the absence of 2-ME, the ORF8 bands displayed multiple ladders (Fig. 3*C*, left). In contrast, the WT and C20A bands were dominantly the dimer size (∼34 kDa) and monomer size (∼18 kDa), respectively (Fig. 3*C*, right). ORF8 has six cysteine residues at amino acids 25, 37, 61, 83, 90, and 102 in addition to the cysteine at amino acid 20, and the crystal structure shows that each monomer has three pairs of intramolecular disulfide bonds (C25–C90, C37–C102, and C61–C83) (Fig. 1*A*) (32). The data indicate that the C20 residue is important for stable homodimer formation through an intermolecular disulfide bond, consistent with the ORF8 structure. In contrast, the other multimers retained intracellularly formed intermolecular disulfide bonds because multimers detected in the C20A mutant display a similar ladder pattern. In addition, the IP data show that the ORF8 dimer is preferentially exported out of cells, although ORF8 dimerization is dispensable for its secretion from cells.

### The ORF8 protein preferentially downregulates cell surface MHC-I molecules

**Figure 4:**
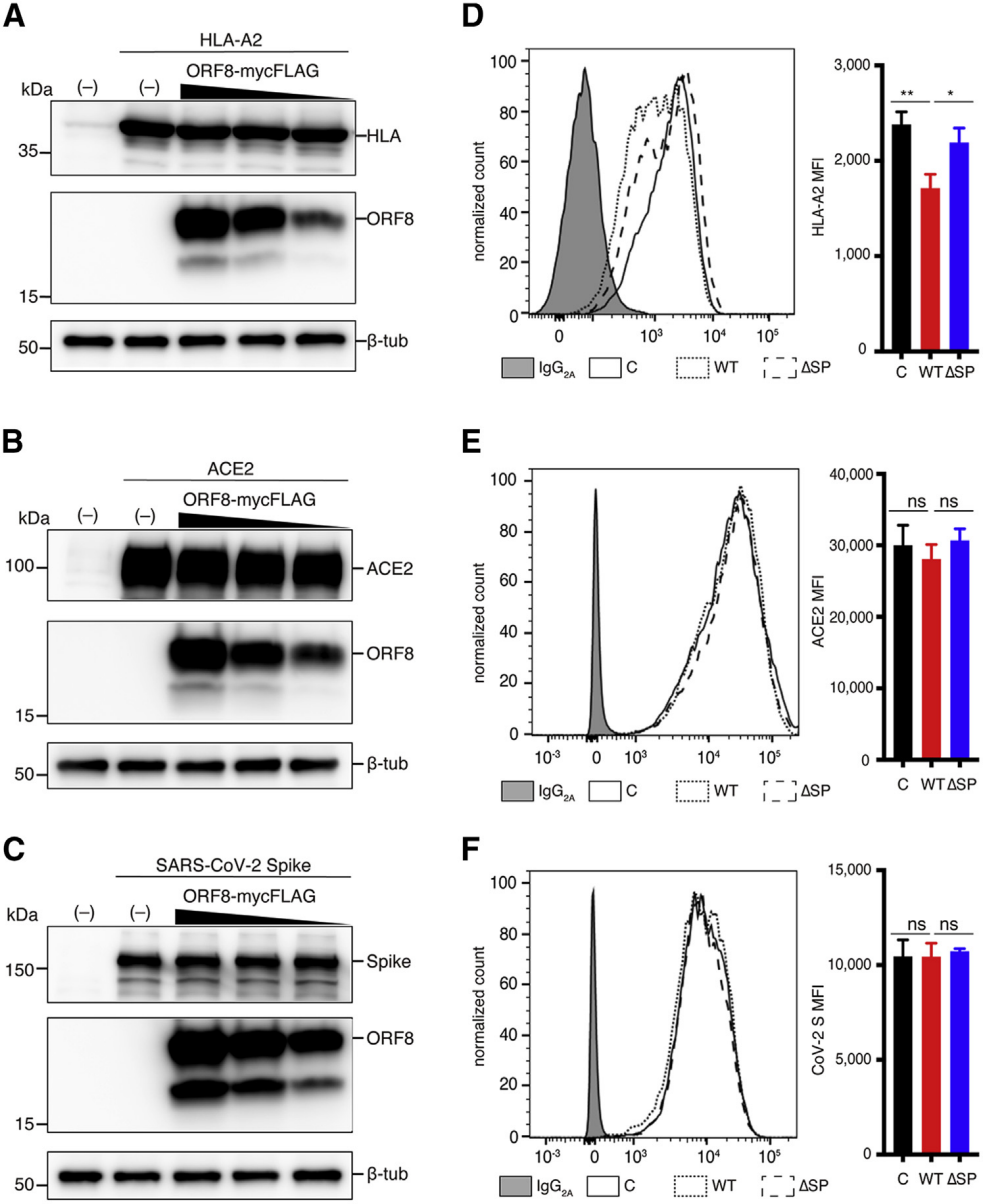
**Effects of SARS-CoV-2 ORF8 on expression and downregulation of HLA-A2, ACE2, and CoV-2 S proteins.***A*–*C*, Western blot analyses of total ORF8 in cells. Briefly, 293T cells were cotransfected with each target expression plasmid (500 ng), (*A*) HLA-A0201-HIS, (*B*)ACE2, or (*C*) CoV-2 S, and different amounts of the expression plasmid encoding ORF8-mycFLAG (CO3) (0, 1500, 1000, and 500 ng). The total plasmid amount for each transfection was normalized with the empty vector. Next, 293T cells were cotransfected with each target plasmid (125 ng), GFP expression plasmid pAcGFP1-N1 (125 ng), plus pQCXIP empty vector control (*C*), ORF8-mycFLAG (WT), or ORF8 ΔSP-mycFLAG (ΔSP)-expressing plasmid (750 ng). *D*–*F*, FACS analyses of cell surface proteins. *D*, endogenous HLA-2 surface expression. 293T cells were cotransfected with pAcGFP1-N1 (125 ng) and the ORF8 expression plasmid (875 ng) for analysis. *E*, ACE2 and (*F*) CoV-2 S expression on the cell surface. Cells were transfected with each expression plasmid and collected at 40 h after transfection for flow cytometry analysis. The mean fluorescence intensities (MFI) of HLA-, ACE2-, and CoV-2 S-positive cells (gated on GFP-positive cells) were shown (n = 3). The bar graphs on the *right* represent the mean ± SD (error bars). Student’s *t* test and one-way ANOVA were used. NS, not significant; ∗*p* < 0.05, ∗∗*p* < 0.005. ORF8, open reading frame 8; SARS-CoV-2, severe acute respiratory syndrome coronavirus 2; WT, wild-type.

Some viruses are known to downregulate antigen-presenting proteins and receptors required for entry expressed on the cell surface upon infection to evade host immunity and to enhance viral replication (34, 35, 36, 37). A previous report showed that HA-tagged ORF8 (ORF8-HA), which is dominantly retained in the cytoplasm, directly interacts with MHC-Ι molecules and leads to their downregulation (30). Because the ORF8-HA was expressed from the ORF8 cDNA, similar to our SF1 ORF8 (Fig. 1), and was ∼15 kDa, which is smaller than the FL glycosylated ORF8 (∼19 kDa), we needed to reevaluate the effect of FL ORF8 on MHC-I downregulation in our ORF8 expression system. Human leukocyte antigen (HLA-A0201 or HLA-A2) was used as the MHC-I molecule. HLA-A2, ACE2, or CoV-2 S was coexpressed with ORF8-mycFLAG in 293T cells, and the total amount of each protein in cells was analyzed by Western blotting. The total amounts of the intracellular HLA-A2 protein were similar in the cells with different expression levels of ORF8 (Fig. 4*A*). Elevated expression of the ORF8 protein did not change the total amounts of ACE2 and CoV-2 S in cells (Fig. 4, *B* and *C*). In contrast, FACS analysis of cell surface levels demonstrated that the level of HLA-A2 was significantly reduced (71.9% ORF8 WT *versus* control) in the presence of ORF8 WT (Fig. 4*D*), although ACE2 and CoV-2 S were not affected by ORF8 WT expression (Fig. 4, *E* and *F*). The levels of HLA-A2, ACE2, and CoV-2 S on the cell surface were similar between the control and the ΔSP (Fig. 4, *D*–*F*). The levels of ACE2 and SARS-CoV-2 S were not downregulated in an ORF8 protein-dependent manner as determined by FACS (Fig. 4, *D*–*F*). These results suggest that ORF8 protein expression induces the downregulation of MHC-I on the cell surface through a nonproteolytic degradation pathway.

### No cytokine-like effects of the ORF8 protein were observed in MDMs

**Figure 5:**
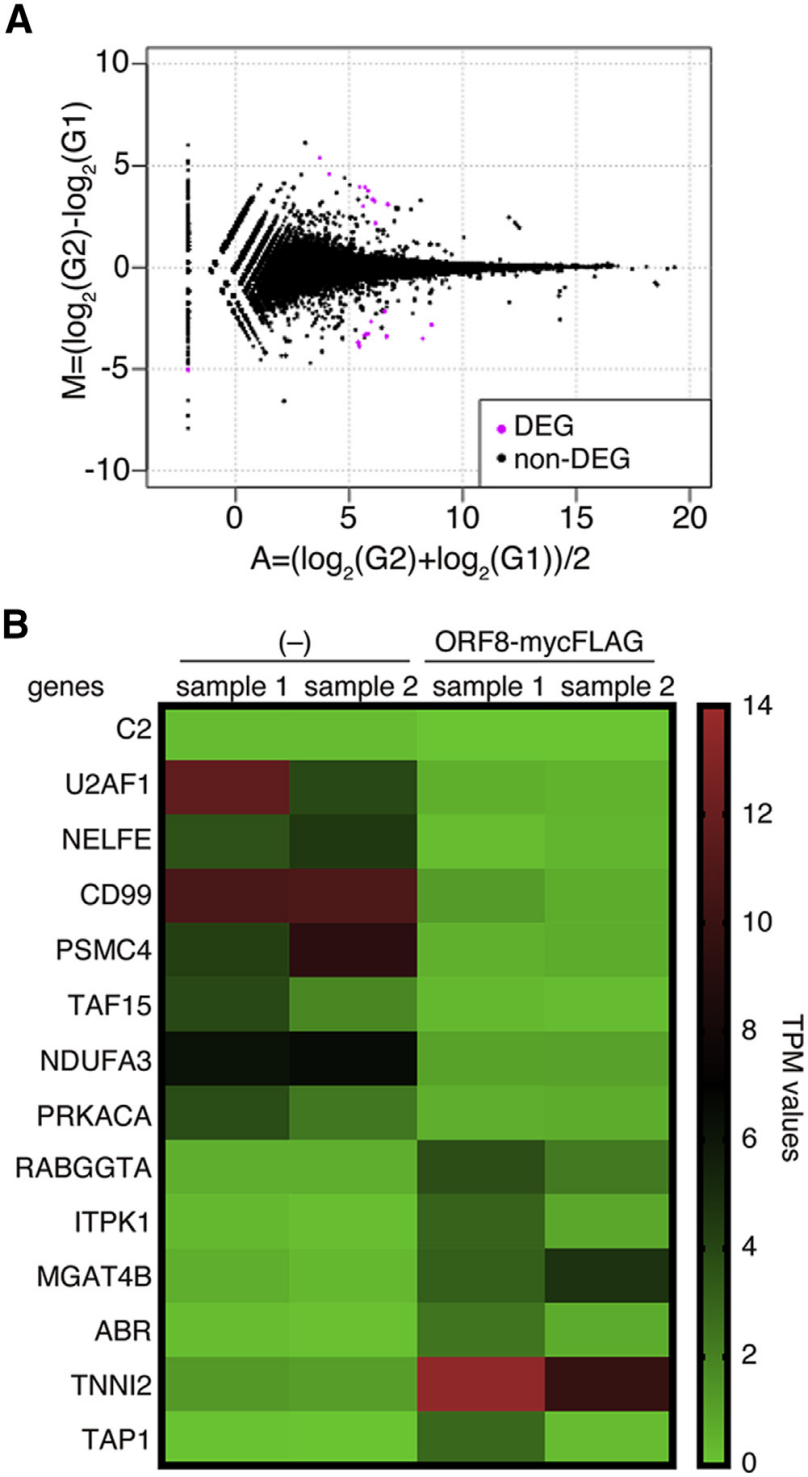
**Expression profiles of mRNAs in ORF8-treated and control MDMs as determined by RNA-Seq.***A*, MA plot of human MDMs treated with the control and ORF8. The differentially expressed genes (DEGs) and nondifferentially expressed genes (non-DEGs) are indicated by *pink* and *black dots*, respectively. A false discovery rate (FDR) <0.05 indicated a significant difference. *B*, heatmap representing the differentially expressed transcripts of nontreated control MDMs (–) and ORF8-treated MDMs. Significantly enriched gene sets with an FDR <0.05 were used. The expression levels, defined as transcripts per million (TPM), are shown. MDM, monocyte-derived macrophage; ORF8, open reading frame 8.

Finally, we assessed whether ORF8 has a stimulatory effect similar to that of cytokines in MDMs because ORF8 is efficiently secreted into the extracellular space as a homodimeric glycoprotein (Fig. 1*E*). In this assay, we used human primary MDM cells that were sensitive to various cytokines. Cell sups with or without ORF8 expression were collected from 293T cell cultures that had been transfected with pQCXIP ORF8-mycFLAG (CO3) or pQCXIP empty vector. The MDM cell culture medium was replaced with the ORF8-containing sup, and the cells were incubated for 2 h. Total RNA was prepared from ORF8-treated cells and subjected to RNA-seq. RNA-seq reads were comparatively analyzed between mock-treated and ORF8-treated MDMs. The results showed that eight genes (C2, U2AF1, NELFE, CD99, PSMC4, TAF15, NDUFA3, and PRKACA) were reduced and six genes (RABGGTA, ITPK1, MGAT4B, ABR, TNNI2, and TAP1) were significantly increased in the ORF8-treated MDMs compared with mock cells (false discovery rate (FDR) < 0.05; Fig. 5, *A* and *B*). Generally, cytokines such as type-I IFNs and IL-6 activate other downstream signals *via* their receptors and induce the expression of a wide variety of genes through master transcription factors, such as members of the signal transducer and activator of transcription (STAT) and/or nuclear factor-kappa B (NF-κB). In contrast, the genes predominantly changed by the addition of secreted ORF8 were not functionally linked to each other. Based on this point, we concluded that there were no cytokine-like effects of the secreted ORF8 protein on MDMs in our assay system.

## Discussion

The goal of this study was to characterize the ORF8 protein to thereby elucidate its biological features in cells because several different biological functions of the molecule have been reported (22, 23, 25, 26, 27, 28, 29, 30, 31). At the beginning of this study, we thought that these different results could be obtained, largely because basic properties of this protein in cells have not yet been fully determined. Therefore, we first set out to establish a protein expression system in cultured cells to prevent the unintended splicing of ORF8 mRNA upon transfection of its expression plasmids. Indeed, the ORF8 cDNA derived from the viral genomic sequence resulted in the expression of a protein with a smaller molecular weight than the theoretical molecular weight, which was caused by unintended splicing at three cryptic splice sites (Fig. 1). Our elaboration of the ORF8 expression plasmids by a combination of codon optimization, mutagenesis, and expression vectors enabled us to express the ORF8 protein at the theoretical molecular size in cells. Interestingly, further analysis of the protein using our system demonstrated that the SARS-CoV-2 ORF8 protein was secreted out of the cell as a homodimeric glycoprotein (Figs. 2 and 3). The 2-ME-sensitive ORF8 multimers partly accumulate in cells. The N-linked glycosylation of ORF8 at N78 was shown to be required for efficient secretion, although intermolecular disulfide bonding at C20 was dispensable for export out of cells. These results suggest that the ORF8 protein plays important roles in the intracellular transport pathway or extracellularly in SARS-CoV-2 infection. In our attempt to identify the biological function of the ORF8 protein, we found that ORF8 induced the downregulation of MHC-I but not of the ACE2 or CoV-2 S proteins on the cell surface (Fig. 4). In terms of secretory proteins, we examined the role of the secreted ORF8 protein as a cytokine-like agonist, but the induction of relevant gene expression in MDMs was not found in our system (Fig. 5).

Previous studies have shown that the ORF8 protein interacts with a variety of host proteins, including IL17RA and factors involved in ER-related degradation and vesicle trafficking (23, 24, 25). In addition, the SARS-CoV-1 ORF8 protein has been reported to antagonize the IFN signaling pathway (26, 28, 29) and ER stress (26, 27). However, consensus in these reports is limited, and progress on SARS-CoV-2 ORF8 protein research remains limited. We hypothesized that some problems would complicate the overexpression system when using cultured cells for the analysis of ORF8 biological functions. Indeed, the molecular weights of the ORF8 proteins in studies reported thus far are not consistent and varied in a range between 14 and 20 kDa (22, 23, 25, 26, 27, 28, 29, 30, 31). Therefore, we first constructed an expression plasmid with a C-terminal mycFLAG tag attached to the cDNA-derived ORF8. Expression of cDNA-derived ORF8 in 293T cells showed that the molecular weight of the expressed ORF8-mycFLAG was slightly smaller than its theoretical value of 15.8 kDa as a glycoprotein (Fig. 1*B*). Our analysis of the mRNA in the transfection revealed that cDNA-derived ORF8 mRNA underwent splicing in the overexpression system (see SF1 ORF8, Fig. 1*B*), which should not naturally occur in the cytoplasm, where SARS-CoV-2 predominantly replicates (Fig. 1*C*). Therefore, we removed cryptic splice sites and optimized the codons and expression vector (retroviral vector) in combination (Fig. 1*B*) and successfully expressed an ORF8 protein of the correct size (approximately 20 kDa including a sugar chain and mycFLAG tag). These results suggest that cryptic splicing events should be considered when cDNA is used to investigate ORF8 functions. Two previous studies showed that a lentiviral vector is efficient for expression of the ORF8 protein at the correct size of 19 kDa (23, 25). This may be partly attributed to the fact that lentiviral vectors are capable of disabling splicing (38). Similarly, in this study, the FL ORF8 was expressed more efficiently with the retroviral vector pQCXIP than with pcDNA3.1 (Fig. 1, *B* and *C*), largely because the pQCXIP has a 5′ long terminal repeat of the murine leukemia virus proviral DNA that potentially enhances the transport of unspliced RNA from nucleus to cytoplasm (39, 40).

The present study showed that ORF8 was efficiently secreted out of the cell as a covalent homodimer with a pair of intermolecular disulfide bonds formed by C20 of each monomer (Fig. 3). Interestingly, the C20A mutation did not affect the glycosylation or extracellular secretion of ORF8 (Fig. 3). This result supports the two previous studies (22, 32). Based on the crystal structure, the intermolecular bonds in the ORF8 dimer are not only intermolecular disulfide bonds, but also residues in the loops connecting β1 and β8 and β3 and β4 that contribute to dimer formation (32). This suggests that the ORF8 protein may feasibly form homodimers, although the C20A mutant does not form intermolecular disulfide bonds. In terms of ORF8 glycosylation modification, our mutational analyses performed with the glycosylase digestion assay revealed that intracellularly accumulated ORF8 had a high mannose-type glycan/hybrid-type glycan, while the secreted ORF8 was conjugated with a complex-type glycan (Fig. 2*B*). Generally, secreted proteins undergo translation and glycosylation (high mannose type) in the ER and are transported to the Golgi apparatus, wherein their glycan structures are mature (41, 42, 43, 44, 45, 46). Intracellular ORF8 with a high mannose-type glycan/hybrid-type glycan was oligomerized (Figs. 2, *B* and *C* and 3*B*). This result suggests that the molecules formed as homodimeric ORF8 may have been transported to the Golgi apparatus, where further maturation of the glycan structure occurred. It is possible that the intracellularly oligomerized ORF8 observed in this study was an unintended byproduct of the virus. Often, when secretory or membrane proteins are expressed, a lack of molecular chaperones or other components in the ER can lead to abnormal protein folding (41, 42, 43, 44, 45, 46). As a consequence, such stacking of abnormal proteins, including oligomeric ORF8, induces ER stress. Currently, which biological functions are associated with the complex glycosylation and dimerization of the ORF8 protein remains unknown, and further studies should be carried out.

Viruses are known to downregulate antigen-presenting proteins and the receptors required for entry expressed on infected cells upon infection of host cells (34, 35, 36, 37). Similar to a previous study on the downregulation of MHC-I cell surface expression by SARS-CoV-2 ORF8 (30), we also observed a slight effect of ORF8 on the downregulation of an MHC-I protein, but not ACE2 and CoV-2 S proteins, in 293T cells (Fig. 4). MHC-I forms a heterodimer with β2 microglobulin (β2m) (47), whereas the ACE2 and CoV-2 S proteins are dimeric and homotrimeric, respectively (48, 49). Because intracellular ORF8 forms disulfide-mediated oligomers in the ER, ORF8 might trap MHC-I molecules by preferentially binding to them or by selectively binding to heterodimeric proteins during oligomer formation. Although Zhang *et al.* (30) reported that MHC-I is degraded *via* autophagy, we could not find an ORF8-dependent MHC-I reduction in cells in our assay system (Fig. 4*A*). While the factor underlying this differential result is unknown, it may be due to the different ORF8 protein contents (SF1 and FL ORF8s) since the molecular weight of the expressed ORF8 protein was dominantly 15 kDa in their report (30). The detailed molecular mechanism of MHC-I downregulation by ORF8 needs to be further investigated.

Some of the secreted viral proteins function as cytokine-like proteins (50, 51). In this study, we also analyzed the stimulatory effects of the secreted ORF8 protein on MDMs as a model, because MDMs are primary cells with high cytokine sensitivity. However, no significant induction of gene expression by the ORF8 protein was observed in MDMs. These results were potentially attributed to the ORF8 protein functioning as an antagonist and not an agonist and/or the inappropriate using of MDMs to identify stimulatory effects. Because SARS-CoV-2 replicates in the lungs and nasal cavity (52, 53), immune cells in mucous membranes and/or tissues should be targeted rather than immune cells in the blood. The followings are limitations of our study regarding the validation of the ORF8 protein we used: (1) the mycFLAG added to the C-terminus may have affected the bioactivity, and (2) the induction time may have been insufficient.

The ORF8-defective SARS-CoV-2 strain (Δ382) found in Singapore has been shown to have a milder pathology, reduced hypoxia, and decreased release of proinflammatory cytokines (11). In contrast, the VOC alpha strain (B.1.1.7) that has an ORF8 truncation induced by a nonsense mutation (Q27∗) and presumably has a nonfunctional ORF8 (20, 21), became prevalent in the winter of 2020 (17, 18, 21). Despite epidemiological evidence that the alpha strain has spread around the world, many severe cases in COVID-19 have been immutably identified worldwide. More recent studies revealed that the VOC omicron variant (B.1.1.529 and the BA lineage) that encodes an intact *orf8* gene displays less pathogenicity (54) and causes mild or even asymptomatic symptoms in infected people (55). This clinical evidence indicates that the *orf8* gene in SARS-CoV-2 does not fully correlate with viral pathogenicity and disease severity. Interestingly, Lin *et al.* have reported that the unglycosylated ORF8 secreted *via* an unconventional pathway is responsible for the COVID-19 cytokine storm through IL-17 receptor-based signaling (31, 56). However, their proposed mechanism seems contradictory to the recent clinical evidence. In addition, the small ORF8 (<14 kDa) was used in the study to assess the effects of the ORF8 protein (31), which corresponds to the size of our observed “SF1 ORF8” (Fig. 1*B*). Therefore, further analyses are required to clarify the ORF8 function using appropriate ORF8 expression systems.

ORF8 is hypothesized to be advantageous for functions within an individual (*e.g.*, viral replication and immune evasion) but neutral or even disadvantageous for transmission (20). Our results revealed that downregulation of MHC-I by ORF8 and the unidentified function of the secreted ORF8 protein might be advantageous, while the intracellular ORF8 protein might be disadvantageous because it induces ER accumulation and ER stress (26, 27) *via* oligomerization. Therefore, the ORF8 protein is considered to be both advantageous and disadvantageous for viruses. Therefore, it is possible that ORF8 is evolving to an optimal state in the individual while balancing these properties.

In conclusion, the SARS-CoV-2 ORF8 protein was found to be secreted extracellularly as a homodimer with a sugar chain of the complex-type glycan. On the other hand, a certain amount of ORF8 is intracellularly accumulated. Therefore, ORF8 may play important roles in the intracellular transport pathway and/or extracellular signaling in SARS-CoV-2 infection. Our study revealed that C20 and N78 play an important role in maintaining the molecular structure of the ORF8 protein in cells. In fact, these two residues of ORF8 in the epidemic strains have been highly conserved according to the Global Initiative on Sharing All Influenza Data (GISAID) (99.9% of 2,156,221 sequences of the FL SARS-CoV-2 genome, registered by December 2021). Therefore, these results might support that these two residues of ORF8 play essential roles in the bioactivity of SARS-CoV-2 infection *in vivo*. Finally, these findings provide an important basis for understanding a novel functional mechanism of the ORF8 protein and for exploring the factors that contribute to human transmission, considering that the *orf8* gene is one of the most rapidly evolving in the betacoronavirus family.

## Experimental procedures

### Cell culture

293T cells and Vero E6 cells (American Type Culture Collection) were maintained in Dulbecco's modified Eagle medium (Sigma-Aldrich) supplemented with 10% fetal bovine serum (FBS), penicillin (100 U/ml) and streptomycin (100 μg/ml) (Thermo Fisher Scientific) in 5% CO_2_ at 37 °C.

### Plasmid construction

To construct the pcDNA 3.1-based ORF8 expression plasmids, we first extracted viral RNA from SARS-CoV-2-infected Vero E6 cells and amplified a DNA fragment of the SARS-CoV-2 *orf8* by nested RT-PCR using the PrimeScript II High Fidelity One Step RT-PCR kit (Takara Bio). The primers for the first PCR (orf8_5'(27841-64)_1(+) and orf8_3'(28327-52)_1(−)) and the second PCR (orf8_ATG(NotI) and orf8_mycFLAG(HindIII)) were used for amplification, and the primer sequences are listed in Table S2. Each amplified DNA fragment containing the sequence for a tandem tag of myc and FLAG peptides (mycFLAG) was inserted between the NotI and HindIII sites of the pcDNA3.1 (–) empty vector (Thermo Fisher Scientific). For pQCXIP ORF8 expression plasmids, a DNA fragment of SARS-CoV-2 *orf8* with a mycFLAG-coding sequence at the 3′ end was generated from pcDNA3.1 ORF8 using PrimeSTAR Max DNA Polymerase (Takara Bio) and a primer set, orf8_ATG(NotI) and Rev_BamHI-FLAG. An amplified fragment was inserted between the NotI and BamHI sites of the pQCXIP empty vector (Takara Bio). The codon-optimized ORF8 expression plasmids were constructed by inserting the synthetic DNA fragment, SARS-CoV-2 *orf8* (purchased from IDT), into pcDNA3.1 (–) and pQCXIP. The synthetic *orf8* fragment was codon-optimized for mammalian cell expression, in which cryptic splice sites were mutated. For the pQCXIP codon-optimized ORF8 ΔSP expression plasmids or pQCXIP codon-optimized ORF8 (no tag) expression plasmids, a DNA fragment was amplified from pQCXIP codon-optimized ORF8 (CO3) by PCR using the primer sets NotI_ATG_F16__o8 and Rev_BamHI-FLAG for ORF8 ΔSP or orf8_ATG(NotI) and BamHI_stop_o8 for ORF8 (no tag) and was then replaced back into the pQCXIP vector. The C-terminal HIS-tagged HLA expression plasmid of pcDNA3.1-HLA-A0201-HIS was constructed by reinserting the HLA cDNA fragment that was amplified by PCR from the pcDNA3.1 HLA-A∗02:01 plasmid (57) using the primer set T7 Promoter and HLA-A0201-C-HIS_XbaI(–). Mutants of the glycosylation site (N78D) and intermolecular disulfide site (C20A) were generated using site-directed mutagenesis with a PrimeSTAR mutagenesis basal kit (Takara Bio). All the sequences of both the insert and the boundary regions of the expression plasmids were verified by conventional DNA sequencing methods. Detailed information about the primers used here is shown in Table S2.

### Transfection

For analysis of the intracellular or extracellular ORF8 protein levels, the ORF8 expression plasmid (2 μg) was transfected into 293T cells in 12-well plates using FuGENE HD (Promega). To analyze the effects of ORF8 on the total amounts of surface proteins, a CoV-2 S expression plasmid (pC-SARS2-S WT) (58), ACE2 expression plasmid (pC-ACE2 WT) (58), and HLA expression plasmid (pcDNA3.1-HLA-A0201-HIS) were used. Each surface protein expression plasmid (500 ng) was cotransfected with different amounts of pQCXIP ORF8(CO3)-mycFLAG WT (0, 1500, 1000, and 500 ng; the plasmid amount was normalized by empty vector) or 1500 ng of the pQCXIP (–) vector control. At 36 h posttransfection, the cell lysates and the culture sup were prepared with Laemmli buffer (Bio-Rad) in the presence or absence of 2.5% (vol/vol) 2-ME. The sup was passed through a 0.45-μm-pore-size filter to remove cellular debris for direct detection by immunoblotting.

For FACS analysis, either pC-SARS2-S WT or pC-ACE2 WT (125 ng), pAcGFP1-N1 (Takara Bio) (125 ng), and 750 ng of pQCXIP ORF8(CO3)-mycFLAG (WT or ΔSP) or pQCXIP (–) were used for cotransfection. To assess the effects of ORF8 expression on endogenous HLA levels on the 293T cell surface, only the pAcGFP1-N1 (125 ng) and ORF8 expression plasmids (875 ng) were transfected. At 40 h posttransfection, the cells were subjected to FACS analyses.

### Western blot analysis

We fractionated 293T cell lysates by SDS-PAGE (12% acrylamide gel) and transferred them onto Immobilon-P membranes (Merck-Millipore). The membranes were first probed with the appropriate primary antibodies. The C-terminal mycFLAG-tagged ORF8 and SARS-CoV-2 ORF8 (no Tag) were detected with an anti-DYKDDDDK (FLAG)-tagged mouse monoclonal antibody (mAb) (1:2000; Fujifilm Wako Pure Chemical Co) and an anti-SARS-CoV-2 ORF8 rabbit polyclonal antibody (1:1000; ABclonal; A20235), respectively. For membrane protein detection, an anti-HIS tag mAb (1:1000; MBL; D291-3) for HIS-tagged HLA, an anti-SARS-CoV/SARS-CoV-2 spike mAb (1A9) (1:1000; GeneTex, Inc; GTX632604) for CoV-2 S, an anti-ACE2 rabbit monoclonal antibody (1:1000; Abcam; ab108252) for ACE2, and an anti-β-tubulin rabbit polyclonal antibody (1:2000; Abcam; ab6046) for β-tubulin (β-tub) were used. The immunoblotted membranes were subsequently incubated with horseradish-peroxidase-conjugated secondary antibodies. A goat anti-mouse IgG antibody (1:20,000; Thermo Fisher Scientific) and a goat anti-rabbit IgG antibody (1:20,000; Thermo Fisher Scientific) were used for FLAG, ORF8, HIS, CoV-2 S, ACE2, and β-tub, respectively. The proteins were visualized by enhanced chemiluminescence using SuperSignal West Dura (Thermo Fisher Scientific) and quantified using an ImageQuant LAS 4000 (GE Healthcare Life Sciences). The molecular size of bands on the immunoblot images were estimated by ImageQuant TL 8.2 (Cytiva) with a molecular weight standard, ECL DualVue Western blotting markers (Cytiva).

### RNA extraction, RT-PCR, and sequencing

Total RNA was isolated from transfected 293T cells using ISOGEN-LS (Nippon Gene) according to the manufacturer’s protocol and treated with RNase-free recombinant DNase I (Takara Bio). The RNA was then reverse-transcribed and amplified by RT-PCR with the PrimeScript One Step RT-PCR Kit Ver. 2 (Takara Bio) using either of two primer sets: T7 Promoter and BGH Reverse (for the pcDNA-based expression) or pQCXIP sequence primer 5′ and pQCXIP sequence primer 3′ (for the pQCXIP-based expression) (Table S2). The products were analyzed on a 2.5% agarose gel and visualized by ethidium bromide staining. The sizes of FL ORF8 and SF1 ORF8 fragments amplified from RNA samples of the pcDNA-based expression were 678 and 552 bps, respectively, while those of the pQCXIP-based expression were 604 and 478 bps, respectively. For sequence determination, the RT-PCR products were purified using a DNA gel extraction kit (Qiagen) and subjected to Sanger sequencing using the primer sets listed in Table S2.

### Immunoprecipitation assay

To enrich the ORF8 proteins in the culture sup, we performed an immunoprecipitation assay as previously described (59, 60). Briefly, 293T cells were transfected with an ORF8 plasmid containing a C-terminal mycFLAG tag. At 36 h posttransfection, the cells and the culture sup were harvested and then lysed in lysis buffer (150 mM NaCl, 1 mM EDTA, 1% Triton X-100, and 50 mM Tris-HCl pH 7.4). For the sup, cell debris was removed using a 0.45 μm filter. The ORF8 protein was immunoprecipitated with anti-FLAG M2 magnetic beads (Sigma-Aldrich) at 4 °C for 1 h. After the beads were washed with lysis buffer, the IP fractions were eluted competitively with 150 μg/ml of the 3× FLAG peptide (Sigma-Aldrich) and then analyzed by Western blotting. To estimate ratio (%) of the secreted ORF8 protein in total, the band intensity of the cell and sup fractions on one blotted membrane was quantified by ImageQuant TL 8.2 (Cytiva). The ratio (%) of the intensity of sup relative to that of sum (cell + sup) and the average value for five independent experiments was calculated.

### Endoglycosidase analysis of ORF8 proteins

For Endo H digestion, eluted fractions of the immunoprecipitants from the cell lysate or culture sup were first diluted with 1× glycoprotein denaturing buffer (0.5% SDS and 40 mM dithiothreitol [DTT]) and boiled at 98 °C for 10 min. The denatured samples were divided into two halves. Half of the sample was diluted with 1× GlycoBuffer 3 (50 mM sodium acetate pH 6.0) (New England Biolabs) and then treated with Endo H (New England Biolabs) at 37 °C for 4.5 h. For PNGase F digestion, the other half of the samples were diluted with 1× GlycoBuffer 2 (50 mM sodium phosphate pH 6.0) and 1% NP-40 and then treated with PNGase F (New England Biolabs) at 37 °C for 4.5 h. Enzyme-treated samples were separated by 12% SDS-PAGE under reducing conditions and analyzed by Western blotting using the anti-FLAG tag mAb.

### FACS analysis

For the analysis of cell surface proteins, cells were collected in phosphate-buffered saline (PBS) containing 1 mM EDTA and washed with PBS containing 2% (wt/vol) FBS. Surface proteins were stained for 30 min with monoclonal antibodies in 2% FBS-PBS on ice. After the cells were washed, the proteins were stained with a mouse F(ab)2 IgG (H + L) APC-conjugated antibody (10 μl/10^6^ cells; R&D Systems; F0101B) and LIVE/DEAD Fixable Viability Dye (0.25 μl/10^6^ cells; Thermo Fisher Scientific; L34955) in 2% FBS-PBS on ice for 30 min. The levels of cell surface proteins were analyzed by flow cytometry using a FACS Canto II (BD Biosciences). The data were analyzed with FlowJo software (BD Biosciences). The following antibodies were used for FACS: anti-HLA-A2 mAb (BB7.2) (1 μg/10^6^ cells; BioLegend; 343302), anti-SARS-CoV/SARS-CoV-2 spike mAb (1A9) (1 μg/10^6^ cells), and anti-ACE2 mAb (1 μg/10^6^ cells).

### Preparation of human MDMs and treatment of ORF8

Human peripheral blood mononuclear cells (PBMCs), obtained by venipuncture from healthy donors, were separated by density centrifugation on Lymphoprep (Veritas) at 800*g* for 30 min. Monocytes were isolated from PBMCs using a Classical Monocyte Isolation Kit (Miltenyi Biotec) or EasySep Human Monocyte Isolation Kit (Veritas). CD14^+^ cells were plated at 5 × 10^5^ cells per well in 6-well plates in RPMI 1640 (Sigma-Aldrich) supplemented with penicillin and streptomycin for 3 h, followed by the addition of 10% FBS (Cytiva) and 10 ng/ml macrophage colony stimulating factor (PeproTech). Adherent cells were cultured for 7 days to enable differentiation into MDMs.

To produce cell sups containing secreted ORF8, at 16 h posttransfection, the culture sup was gently washed three times and replaced with fresh RPMI 1640 containing penicillin, streptomycin, and 10% FBS, followed by an additional 24 h incubation. The sups were harvested and clarified by centrifugation at 500*g* for 10 min. The culture sup was passed through a 0.45-μm-pore-size filter to remove cellular debris. The cell sup containing the secreted ORF8 protein was added to MDMs and incubated at 37 °C for 2 h.

### RNA-seq

With 300 ng of total RNA from MDMs, an RNA-seq library was constructed using the MGIEasy RNA Directional Library Prep Set and MGIEasy Circularization Kit (MGI) according to the manufacturer’s instructions. The library was loaded on a DNBSEQ-G400RS sequencing flow cell with a DNBSEQ-G400RS high-throughput sequencing kit (MGI) and run on DNBSEQ-G400 (2 × 100 bp). Sequencing data from DNBSEQ-G400 were analyzed using standard protocols. Briefly, raw reads were preprocessed using cutadapt 1.9.1 (61) and sickle 1.33 (https://github.com/najoshi/sickle) and then mapped to the human genome (GRCh38) using HISAT2 version 2.2.0 (62) and Samtools version v.1.10 (63). The gene levels of the obtained read counts were estimated with featureCounts version 2.2.0 (64).

## Data availability

RNA seq data are archived at the BioProject of the DNA Data Bank of Japan (DDBJ) (https://www.ddbj.nig.ac.jp/index-e.html) under accession number PRJDB12786 (Dataset title, the effects of the SARS-CoV-2 ORF8 secretory glycoprotein on human primary monocyte-derived macrophages (MDMs)).

## Supporting information

This article contains supporting information.

## Conflict of interest

The authors declare that there are no conflicts of interest with the contents of this article.
