# Supplementary material for: SARS-CoV-2 accessory protein ORF8 is secreted extracellularly as a glycoprotein homodimer
Source: J Biol Chem. 2022 Feb 11;298(3):101724. doi: 10.1016/j.jbc.2022.101724 (PMC8832879; doi:10.1016/j.jbc.2022.101724)
Supplement: Supplemental Tables S1 and S2 [file mmc1.docx]

**Table S1.** Nucleotide coding sequences for the ORF8s used in this study

| Name | Sequence (5' > 3') |
| --- | --- |
| ORF8-mycFLAG cDNA | ATGAAATTTCTTGTTTTCTTAGGAATCATCACAACTGTAGCTGCATTTCACCAAGAATGTAGTTTACAGTCATGTACTCAACATCAACCATATGTAGTTGATGACCCGTGTCCTATTCACTTCTATTCTAAATGGTATATTAGAGTAGGAGCTAGAAAATCAGCACCTTTAATTGAATTGTGCGTGGATGAGGCTGGTTCTAAATCACCCATTCAGTACATCGATATCGGTAATTATACAGTTTCCTGTTCACCTTTTACAATTAATTGCCAGGAACCTAAATTGGGTAGTCTTGTAGTGCGTTGTTCGTTCTATGAAGACTTTTTAGAGTATCATGACGTTCGTGTTGTTTTAGATTTCATCGAACAAAAACTCATCTCAGAAGAGGATCTGGATTACAAGGATGACGACGATAAGTAG |
| ORF8-mycFLAG CO1 | ATGAAATTTCTTGTTTTCTTAGGAATCATCACAACAGTCGCGGCTTTTCATCAAGAATGCTCTTTGCAGTCTTGTACACAGCATCAACCTTATGTCGTCGATGATCCTTGCCCGATTCACTTTTACAGTAAATGGTATATCAGGGTAGGGGCCCGAAAAAGCGCACCATTGATTGAATTGTGTGTGGACGAGGCAGGGAGCAAAAGTCCGATTCAATACATTGACATCGGTAATTATACTGTCAGTTGTTCCCCCTTTACGATCAACTGCCAAGAACCCAAACTGGGGAGCTTGGTGGTCCGATGTTCCTTCTATGAAGATTTTCTTGAGTATCACGATGTTCGTGTTGTTTTAGATTTCATCGAACAAAAACTCATCTCAGAAGAGGATCTGGATTACAAGGATGACGACGATAAGTAG |
| ORF8-mycFLAG CO2 | ATGAAATTTCTTGTTTTCTTAGGAATCATCACAACAGTCGCGGCTTTTCATCAAGAATGCTCTTTGCAGTCTTGTACACAGCATCAACCTTATGTCGTCGATGATCCTTGCCCGATTCACTTTTACAGCAAGTGGTACATTCGCGTAGGGGCCCGAAAAAGCGCACCATTGATTGAATTGTGTGTGGACGAGGCAGGGAGCAAAAGTCCGATTCAATACATTGACATCGGCAACTACACTGTCTCTTGTTCCCCCTTTACGATCAACTGCCAAGAACCCAAACTGGGGAGCTTGGTGGTCCGATGTTCCTTCTATGAAGATTTTCTTGAGTATCACGATGTTCGTGTTGTTTTAGATTTCATCGAACAAAAACTCATCTCAGAAGAGGATCTGGATTACAAGGATGACGACGATAAGTAG |
| ORF8-mycFLAG CO3 | ATGAAATTTCTTGTTTTCTTAGGAATCATCACAACAGTCGCGGCTTTTCATCAAGAATGCTCTTTGCAGTCTTGTACACAGCATCAACCTTATGTCGTCGATGATCCTTGCCCGATTCACTTTTACAGCAAGTGGTATATTCGCGTAGGGGCCCGAAAAAGCGCACCATTGATTGAATTGTGTGTGGACGAGGCAGGGAGCAAAAGTCCGATTCAATACATTGACATCGGCAACTACACTGTCTCTTGTTCCCCCTTTACGATCAACTGCCAAGAACCCAAACTGGGGAGCTTGGTGGTCCGATGTTCCTTCTATGAAGATTTTCTTGAGTATCACGATGTTCGTGTTGTTTTAGATTTCATCGAACAAAAACTCATCTCAGAAGAGGATCTGGATTACAAGGATGACGACGATAAGTAG |
| ORF8 SF1 | ATGAAATTTCTTGTTTTCTTAGGAATCATCACAACTGTAGCTGCATTTCACCAAGAATGTAGTTTACAGTCATGTACTCAACATCAACCATATGTAGTTGATGACCCGTGTCCTATTCACTTCTATTCTAAATGGTATATTAGAGTAGGAGCTAGAAAATCAGCACCTTTAATTGAATTGTGCGTGGATGAGGCTGGTTCTAAATCACCCATTCAGTACATCGATATCGATTTCATCTAA |
| ORF8 SF2 | ATGAAATTTCTTGTTTTCTTAGGAATCATCACAACAGTCGCGGCTTTTCATCAAGAATGCTCTTTGCAGTCTTGTACACAGCATCAACCTTATGTCGTCGATGATCCTTGCCCGATTCACTTTTACAGTAAATGATTTCATCTAA |

**Table. S2. Oligonucleotides used in this study**

| Primer Name | DNA sequence (5' > 3') |
| --- | --- |
| orf8_ATG(NotI) | CAGTGGCGGCCGCCACCATGAAATTTCTTGTTTTCTTAGGAATCATCAC |
| orf8_mycFLAG(HindIII) | TTCCAAGCTTACTACTTATCGTCGTCATCCTTGTAATCCAGATCCTCTTCTGAGATGAGTTTTTGTTCGATGAAATCTAAAACAACACGAA |
| Rev_BamHI-FLAG | GAGAGGATCCCTACTTATCGTCGTCATCCTTG |
| NotI_ATG_F16__o8 | GAGAGCGGCCGCCACCATGTTTCATCAAGAATGCT |
| BamHI_stop_o8 | GAGAGGATCCTTACTAGATGAAATCTAAAACAACA |
| Fwd_o8_N78Dv2 | ATCGGCGACTACACTGTCTCTTGTTCCCCCTTTAC |
| Rev_o8_N78Dv2 | AGTGTAGTCGCCGATGTCAATGTATTGAATCGGAC |
| Fwd_o8_C20Av2 | TCAAGAAGCCTCTTTGCAGTCTTGTACACAGCATC |
| Rev_o8_C20Av2 | CAAAGAGGCTTCTTGATGAAAAGCCGCGACTGTTG |
| orf8_donor_mutav3_Fwd | CAACTACACTGTCTCTTGTTCCCCCTTTACGATCA |
| orf8_donor_mutav3_Rev | GAGACAGTGTAGTTGCCGATGTCAATGTATTGAAT |
| orf8_donor2_mutav2_Fwd | CAAGTGGTACATTCGCGTAGGGGCCCGAAAAAGCGCACCATTGATTG |
| orf8_donor2_mutav2_Rev | GCGAATGTACCACTTGCTGTAAAAGTGAATCGGGCAAGGATCATCGACGAC |
| orf8_donor2_mutav3_Fwd | CAAGTGGTATATTCGCGTAGGGGCCCGAAAAAGCGCACCATTGATTG |
| orf8_donor2_mutav3_Rev | GCGAATATACCACTTGCTGTAAAAGTGAATCGGGCAAGGATCATCGACGAC |
| T7 Promoter | TAATACGACTCACTATAGGG |
| BGH Reverse | TAGAAGGCACAGTCGAGGCTG |
| pQCXIP sequence primer 5' | ACGCCATCCACGCTGTTTTGACCT |
| pQCXIP sequence primer 3' | AAGCGGCTTCGGCCAGTAACGTTA |
| HLA-A0201-C-HIS_XbaI(–) | GTGTTTCTAGATTATCAATGGTGATGGTGATGATGACCGGTCACTTTACAAGCTGTGAGAGACACATCAG |
