# Supplementary material for: SARS-CoV-2 accessory protein ORF8 is secreted extracellularly as a glycoprotein homodimer
Source: J Biol Chem. 2022 Feb 11;298(3):101724. doi: 10.1016/j.jbc.2022.101724 (PMC8832879; doi:10.1016/j.jbc.2022.101724)
Supplement: Supplemental Figure S1 [file mmc2.pdf]

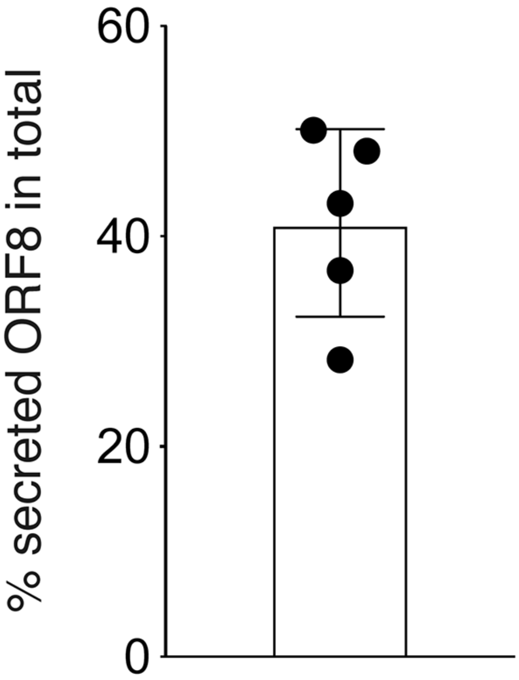

Figure S1. Ratio (%) of secreted ORF8 protein in total. The FLAG-tagged ORF8 proteins were immunoprecipitated from each fraction of the transfected cell and supernatant with anti-FLAG mAb beads. The IP fractions were analyzed by immunoblotting using anti-FLAG mAb. The band intensity of the cell and the supernatant was quantified. The ratio (%) was determined by dividing the supernatant level by the sum (the cell and supernatant). The bar graph represents the mean ± SD (error bars) from five independent assays.
